# Three Novel Calreticulin Mutations in Two Turkish Patients

**DOI:** 10.4274/tjh.2017.0146

**Published:** 2017-12-01

**Authors:** Veysel Sabri Hançer, Hüseyin Tokgöz, Serkan Güvenç, Ümran Çalışkan, Murat Büyükdoğan

**Affiliations:** 1 İstinye University Faculty of Medicine, Department of Medical Genetics, İstanbul, Turkey; 2 Selçuk University Meram Faculty of Medicine, Department of Pediatric Hematology, Konya, Turkey; 3 Batman District State Hospital, Clinic of Hematology Batman, Turkey; 4 Necmettin Erbakan University Meram Faculty of Medicine, Department of Pediatrics, Konya, Turkey

**Keywords:** Essential thrombocythemia, Primary myelofibrosis, Calreticulin

## To The Editor,

Calreticulin (CALR) mutations were first identified exclusively in JAK2-MPL-negative essential thrombocythemia (ET) and primary myelofibrosis (PMF) at a rate of 60%-88%, accounting for 1/4 to 1/3 of all patients with ET and PMF [1,2,3]. As of today, more than 55 different types of mutations have been reported. The two most common mutations accounting for 85% of mutated cases are either a 52-bp deletion (type 1; c.1099_1150del; L367fs*46; 44%-53% of cases) or a 5-bp insertion (type 2; c.1154_1155insTTGTC; K385fs*47; 32%-42% of cases). The remaining 15% include various other infrequent mutations that are often unique or found in only a few patients [4,5].

**Figure 1:**
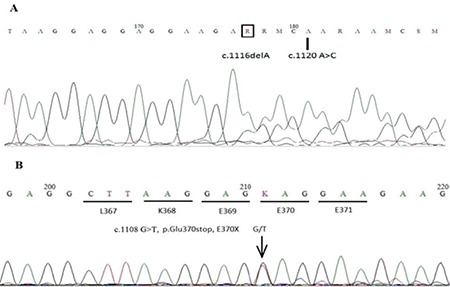
A) Inclusion bodies in neutrophils and macrothrombocyte; B), C) inclusion bodies in neutrophils; D) peripheral blood smear after treatment.

Here we present three CALR mutations in two patients with PMF and ET that have not been reported before as shown in Figure 1. Known CALR mutations and BCR-ABL, JAK-2 V617F, and MPL 515L/K test results were found to be negative.

**Patient 1:** The patient was a 46-year-old man with low back pain. Magnetic resonance imaging scanning of the lumbosacral region revealed sacroiliitis on the left side and he was referred to a rheumatologist for further investigations. Anemia (Hb: 10.8 g/dL) and thrombocytosis (700x10^9^/L) with a high lactate dehydrogenase level (351 U/L) were found in initial tests. The other tests for a possible rheumatologic disease, including human leukocyte antigen-B27, were all negative when the patient was seen. Physical examination was almost normal with no sign of organomegaly. Spleen size was also normal in the abdominal ultrasound. The peripheral blood smear showed dacrocytes, occasional myelocytes (1%), and metamyelocytes (1%). The bone marrow biopsy showed diffuse grade 3-4 reticulin fibrosis with atypical proliferation of megakaryocytes and increased cellularity consistent with PMF.

**Patient 2:** A 9-year-old pediatric patient with thrombocytosis (2800x10^9^/L) was identified in a routine check-up. Physical examination was normal except for mild splenomegaly. Complete blood count revealed increased platelet count (2800x10^9^/L) with normal hemoglobin and leukocyte count. Platelets were very abundant and clustered in the peripheral blood smear. Bone marrow aspiration and biopsy examinations showed tri-lineage hematopoiesis with an increased number and clusters of megakaryocytes without fibrosis, which is consistent with ET. She had persistently elevated platelet counts ranging between 2000x10^9^/L and 2800x10^9^/L without any evidence of reactive/secondary thrombocytosis such as infections, medicine, autoimmune disorders, neoplasms, trauma, surgery, or hematological disorders such as iron deficient anemia, chronic hemolytic situations, and acute hemorrhages.

Genomic DNA was extracted from whole blood, exon 9 of the CALR gene was amplified by polymerase chain reaction, and then the amplified fragments were sequenced. All nucleotide numbers refer to the wild-type cDNA sequence of CALR (NM_004343) as reported in Ensembl. Here we report three new CALR mutations [1-bp deletion; c.1116delA (D373fs*57) and c.1120 A>C] in the same patient with PMF and c.1108 G>T in a patient with ET. We performed germline testing from the cheek epithelium and both patient samples were confirmed as wild-type CALR. These novel mutations occurred and changed the amino acid sequence of the C domain amino acid residues, which will interfere with the calcium-binding capacity of the molecule. It is important to determine the type of mutation. Type 2-like CALR mutations are mainly associated with an ET phenotype, low risk of thrombosis, and indolent clinical course, while type 1-like mutations are mainly associated with a myelofibrosis phenotype and a high risk of progression from ET to myelofibrosis. The identification of new CALR mutations will improve our understanding of the pathophysiology of myeloproliferative neoplasms.

## References

[1] Klampfl T, Gisslinger H, Harutyunyan AS, Nivarthi H, Rumie E, Milosevic JD, Them NC, Berg T, Gisslinger B, Pietra D, Chen D, Vladimer GI, Bagienski K, Milanesi C, Casetti IC, Sant’Antonio E, Ferretti V, Elena C, Schischlik F, Cleary C, Six M, Schalling M, Schönegger A, Bock C, Malcovati L, Pascutto C, Superti-Furga G, Cazzola M, Kralovics R (2013). Somatic mutations of calreticulin in myeloproliferative neoplasms. N Engl J Med.

[2] Fu R, Fu L, Yang R (2013). Paediatric essential thrombocythaemia: clinical and molecular features, diagnosis and treatment. Br J Haematol.

[3] Guglielmelli P, Nangalia J, Green AR, Vanucchi AM (2014). CALR mutations in myeloproliferative neoplasms: hidden behind the reticulum. Am J Hematol.

[4] Tefferi A, Wassie EA, Guglielmelli P, Nangat N, Belachew AA, Lasho TL, Finke C, Ketterling RP, Hanson CA, Pardanani A, Wolanskyj AP, Maffioli M, Casalone R, Pacilli A, Vannucchi AM, Passamonti F (2014). Type 1 versus Type 2 calreticulin mutations in essential thrombocythemia: a collaborative study of 1027 patients. Am J Hematol.

[5] Giona F, Teofili L, Capodimonti S, Laurino M, Martini M, Marzella D, Palumbo G, Diverio D, Foà R, Larocca LM (2014). CALR mutations in patients with essential thrombocythemia diagnosed in childhood and adolescence. Blood.

